# An Investigation into the PVA:MC:NH_4_Cl-Based Proton-Conducting Polymer-Blend Electrolytes for Electrochemical Double Layer Capacitor (EDLC) Device Application: The FTIR, Circuit Design and Electrochemical Studies

**DOI:** 10.3390/molecules27031011

**Published:** 2022-02-02

**Authors:** Shujahadeen B. Aziz, Elham M. A. Dannoun, Mohamad A. Brza, Niyaz M. Sadiq, Muaffaq M. Nofal, Wrya O. Karim, Sameerahl I. Al-Saeedi, Mohd F. Z. Kadir

**Affiliations:** 1 Hameed Majid Advanced Polymeric Materials Research Lab., Physics Department, College of Science, University of Sulaimani, Qlyasan Street, Kurdistan Regional Government, Sulaimani 46001, Iraq; niyaz.sadiq@univsul.edu.iq; 2 Department of Civil Engineering, College of Engineering, Komar University of Science and Technology, Kurdistan Regional Government, Sulaimani 46001, Iraq; 3 Associate Chair of the Department of Mathematics and Science, Woman Campus, Prince Sultan University, Riyadh 11586, Saudi Arabia; elhamdannoun1977@gmail.com; 4 Medical Physics Department, College of Medicals & Applied Science, Charmo University, Chamchamal, Sulaimania 46023, Iraq; mohamad.brza@gmail.com; 5 Department of Mathematics and Science, Prince Sultan University, Riyadh 11586, Saudi Arabia; muaffaqnofal69@gmail.com; 6 Chemistry Department, College of Science, University of Sulaimani, Qlyasan Street, Kurdistan Regional Government, Sulaimani 46001, Iraq; wrya.karim@univsul.edu.iq; 7 Department of Chemistry, College of Science, Princess Nuourah Bint Abdulrahman University, Riyadh 11586, Saudi Arabia; sialsaeedi@pnu.edu.sa; 8 Centre for Foundation Studies in Science, University of Malaya, Kuala Lumpur 50603, Malaysia; mfzkadir@um.edu.my

**Keywords:** polymer blending, ammonium salt, FTIR, EIS, circuit design, TNM, LSV, CV

## Abstract

In this report, the preparation of solid polymer electrolytes (SPEs) is performed from polyvinyl alcohol, methyl cellulose (PVA-MC), and ammonium chloride (NH_4_Cl) using solution casting methodology for its use in electrical double layer capacitors (EDLCs). The characterizations of the prepared electrolyte are conducted using a variety of techniques, including Fourier transform infrared spectroscopy (FTIR), electrical impedance spectroscopy (EIS), cyclic voltammetry (CV), and linear sweep voltammetry (LSV). The interaction between the polymers and NH_4_Cl salt are assured via FTIR. EIS confirms the possibility of obtaining a reasonably high conductance of the electrolyte of 1.99 × 10^−3^ S/cm at room temperature. The dielectric response technique is applied to determine the extent of the ion dissociation of the NH_4_Cl in the PVA-MC-NH_4_Cl systems. The appearance of a peak in the imaginary part of the modulus study recognizes the contribution of chain dynamics and ion mobility. Transference number measurement (TNM) is specified and is found to be (*t_ion_*) = 0.933 for the uppermost conducting sample. This verifies that ions are the predominant charge carriers. From the LSV study, 1.4 V are recorded for the relatively high-conducting sample. The CV curve response is far from the rectangular shape. The maximum specific capacitance of 20.6 F/g is recorded at 10 mV/s.

## 1. Introduction

In electrochemical energy devices, one of the fundamental parts is the polymer electrolyte (PE). Among the electrochemical properties of solid materials, ionic conduction has to be highly focused on ion-conducting materials to be utilized in the energy storage and conversion devices [1]. To our knowledge, PEs appear as materials of interest that possess the required conductivity, as well as electrochemical stability [1,2,3]. For example, dimensional stability, durability, a relatively wide potential window (usually above 1.5 V), and its eco-friendly nature are all properties of these materials [4]. The characterizations of PEs are highly required because of its utilization in multidisciplinary fields, such as electrochemistry, polymer science, organic chemistry, and inorganic chemistry [5]. As a potential alternative to liquid electrolytes, solid polymer electrolytes (SPEs) have received considerable interest as the safest alternative [6].

SPEs are superior to conventional liquid electrolytes, for instance, their lack of leakage, low flammability, good flexibility, safety, stability, and their compatible contact with the electrode [7]. From the large-scale point of view, SPEs have to have both high DC ionic conductivity, reaching σ_dc_ ≥ 10^−5^ S/cm, and enough mechanical strength, in addition to high electrochemical and thermal stabilities [8]. To date, solid biopolymer electrolytes (SBEs) have utilized worldwide, especially in electrochemical devices [9]. In addition, biopolymer-based electrolytes exceed the outstanding drawbacks of synthetic polymer electrolytes, regarding their cost and environmentally friendly nature [10].

From an application point of view, poly (vinyl alcohol) (PVA) is one of polymeric materials which is desirable because of its solubility in water, as well as having a superior charge storage capability, a relatively high dielectric strength, and a desired optical property [11]. The polar polymer of the mentioned polymer belongs to the existence of polar groups enriched in electrons that enables them to coordinate with the cation or surface groups of the fillers. This makes the polymer of interest capable for stabilizing a homogeneous nanocomposite [5,12]. From a structural view, PVA consists of a carbon chain backbone containing hydroxyl groups which are able to form hydrogen bonding, leading to the development of the polymer nanocomposite. Therefore, this polymer is eligible for its integration into the multilayer coating of organic solar cells, owing to its capability to create a barrier to oxygen, and its good transparency [13]. The properties of the blended polymers are superior to the single polymer ones [14]. Polymer blends are physically mixed polymers that are structurally different, interacting through secondary forces, i.e., no covalent bonding, and are miscible at the molecular levels [15]. It was previously confirmed that the conductivity of a PE can be enhanced if the host is a blend of two polymers. Polymer blending can provide the guarantee of structural stability [16]. Ramesh and Liew [17] documented an increased ionic conductivity after the poly(methyl methacrylate) (PMMA)- poly(vinyl chloride) (PVC)-based PE was blended with lithium bis(trifluoromethane) sulfonamide (LiTFSI) [17]. In industrial and commercial applications, polymer blends are viable products, owing to their exceptional properties that are better than those of the component polymers [18]. A comprehensive understanding of the conduction mechanisms within polymer-based electrolytes lead to the knowledge of the nature of ion transport. There are attempts to improve the conductivity of PEs using various salts and polymers at room temperature, which are still in progress. Both the charge transport processes and ion relaxation of SPEs have been the most intensively focused on as a part of condensed matter physics [5,19].

In this work, NH_4_Cl salt at different concentrations were added to the PVA:MC blend of electrolytes to increase the ionic conductivity and enhance the electrochemical properties. M Muthuvinayagam and K Sundaramahalingam [20] prepared proton-conducting SPEs comprising a poly(ethylene oxide) (PEO):polyvinylpyrrolidone (PVP) blend with ammonium nitrate (NH_4_NO_3_). The authors obtained the ionic conductivity of a 6.39 × 10^−5^ S/cm for 12 wt% NH_4_NO_3_ doped polymer system at room temperature. Aziz et al. [21] fabricated glycerolized polyvinyl alcohol (PVA):chitosan PEs. The authors obtained a conductivity of 1.37 × 10^−4^ S/cm and they showed that their samples are eligible for applications in electrochemical energy storage devices. Wang et al. [22] have added NH_4_Cl to the sol–gel-derived ZnO precursor to tune the band structure and electron mobility of the electron transport layer (ETL), as well as decreasing the ZnO film work function and tuning the perovskite film surface morphology. Oku et al. [23] showed the influences of loading NH_4_Cl, by an air blowing process, on CH_3_NH_3_PbI_3_(Cl) perovskite solar cells. They fabricated CH_3_NH_3_PbI_3_(Cl) solar cells comprising different concentrations of NH_4_Cl. The authors found that the voltage-current density characteristic of the cell was increased by loading a suitable NH_4_Cl amount, as well as air blowing, which improved the efficiency of the photoconversion to 14%.

In this work, the relaxation mechanism and the conductivity associated with ion motion within a series of ion-conducting PE, based on PVA:MC, using AC impedance spectroscopy, was addressed. The technique can be used for measuring the dielectric and electrical properties of nanocomposites. Moreover, the current study involved the adoption of a comprehensive understanding of the relaxation and ion transport processes within nanocomposites. The results showed the eligibility of the system to be utilized as a separator in electrochemical devices. The PE under study can be used as a membrane, containing ions.

## 2. Results and Discussion

### 2.1. FTIR Study

**Figure 1:**
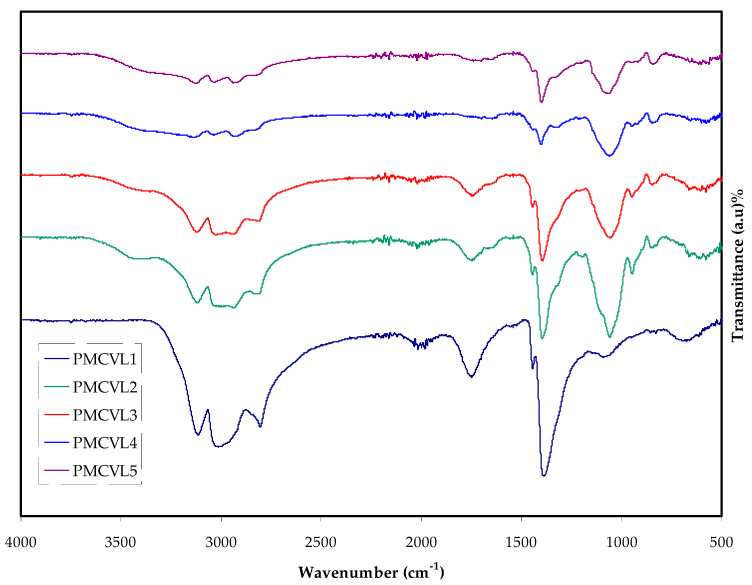
FTIR spectra at a wavenumber between 500 cm^−1^ and 4000 cm^−1^ for PMCVL1, PMCVL2, PMCVL3, PMCVL4, and PMCVL5 electrolyte films.

FTIR spectroscopy is a valuable scheme which can be used to distinguish the degree to which ions and atoms interact in PE systems. The verification of this interaction is acquired by observing the changes in the vibrational bands in the PE [24]. The FTIR absorption bands of the PVA:MC:NH_4_Cl system is seen in Figure 1. Earlier studies documented that any fluctuation and decrease, or increase, in the FTIR absorption bands was evidence for the occurrence of interactions among the salt and polymers [25]. C–H rocking is considered to relate to the absorption bands at 836 cm^−1^ and 839 cm^−1^ [24]. Absorption bands at 1418 cm^−1^ and 1424 cm^−1^ are consistent with −CH_2_ wagging [26]. A strong band at 3318 cm^−1^ and 3339 cm^−1^ is ascribed an O-H stretching vibration [27]. This high-intensity absorption band may be attributable to the strong intra- and inter-hydrogen bonding [28]. Adding NH_4_Cl to the sample provided a shift in the position of this absorption band and its intensity reduction. A relatively weaker absorption peak at 1638 cm^−1^ is ascribed to C = O, with a stretching vibration of the acetate group as a residual part of PVA [26]. At 2921 cm^−1^, the C–H asymmetric vibration band associated with the stretching vibration appeared [27]. When the salt concentration increased, a shift in the location and an intensity reduction of the band were observed. A stretching vibration of the absorption peak at 1079 cm^−1^ is observed in Figure 1, associated with —C—O— in pure PVA [29].

### 2.2. EIS Study

**Figure 2:**
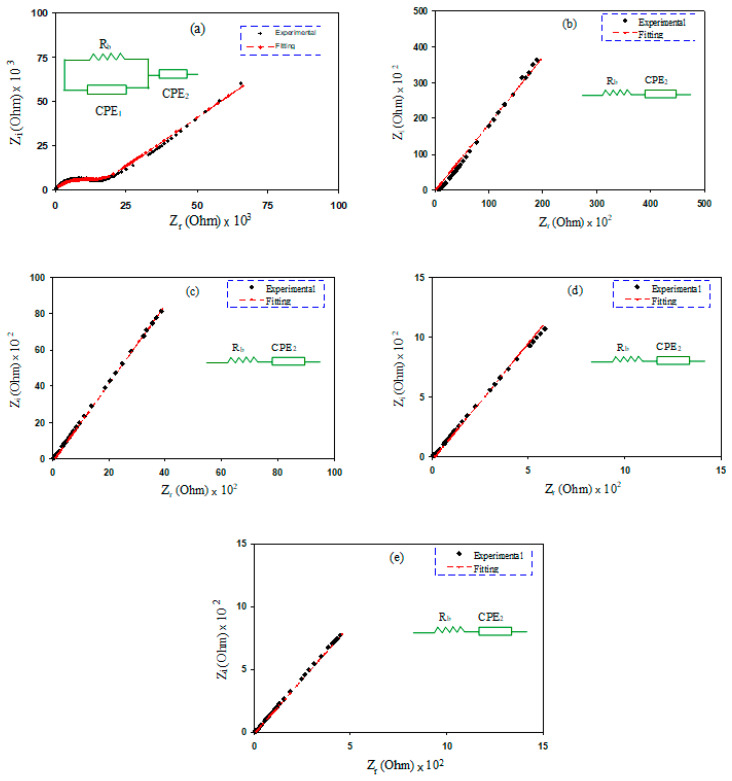
EIS plots for (**a**) PMCVL1, (**b**) PMCVL2, (**c**) PMCVL3, (**d**) PMCVL4, and (**e**) PMCVL5.

EIS is an informative method that is often used to deal with the electrical properties of materials and the interfaces with electronically conducting electrodes through a broad range of temperatures and frequencies. EIS can be used to distinguish between the bulk electrolyte and electrode contributions of charge carriers within the samples. From this technique, one can study the dielectric relaxation and ion conduction processes within the PEs [30]. The characteristic feature of complex impedance spectroscopy (CIS) is the response of the system that is represented by an equivalent circuit [31,32]. In terms of the physics point of view, the resistance (*R*) reflects the dissipated component of the dielectric response, and the capacitance (*C*) describes the extent of the charge stored within the dielectric materials [33,34]. Among the outputs of EIS, the imaginary part of the impedance versus the real part is an outstanding one. For such a system (Figure 2a), it consists of a semicircle at the high-frequency region and a spike at the low-frequency region. The EEC in Figure 2a is represented by a parallel combination of the constant phase element one (*CPE*_1_), bulk resistance (*R_b_*), and in a series with the constant phase element two (*CPE*_2_). The real capacitor replaces the *CPE* to represent the depressed semicircle [35,36]. The impedance of *Z_CPE_* can be expressed in the following mathematical expression [37,38]:

(1) $$Z_{CPE}=\frac{1}{Q\omega^{n}}e^{-j\frac{\pi }{2}n}=\frac{1}{Q\omega^{n}}\left[\cos\left(\frac{\pi n}{2}\right)-j\sin\left(\frac{\pi n}{2}\right)\right],0\leq n\leq 1$$

where *Q* is the *CPE* capacitance, *ω* is the angular frequency, and *n* stands for the deviation of the response from the vertical axis in the CIS plots. Thus, the values of *Z_r_* and *Z_i_* linked with the EEC (insets of Figure 2a) and are shown below:

(2) $$Z_{r}=\frac{R_{1}+R_{1}^{2}Y_{1}\omega^{n1}\cos(\pi n_{1}/2)}{1+2R_{1}Q_{1}\omega^{n_{1}}\cos(\pi n_{1}/2)+R_{1}^{2}Q_{1}^{2}\omega^{2n_{1}}}+\frac{\cos(\pi n_{2}/2)}{Q_{2}\omega^{n_{2}}}$$

(3) $$Z_{i}=\frac{R_{1}^{2}Q_{1}\omega^{n_{1}}\sin(\pi n_{1}/2)}{1+2R_{1}Q_{1}\omega^{n_{1}}\cos(\pi n_{1}/2)+R_{1}^{2}Q_{1}^{2}\omega^{2n_{1}}}+\frac{\sin(\pi n_{2}/2)}{Q_{2}\omega^{n_{2}}}$$

All elements of the EEC for each sample are shown in Table 1. It is generally seen that the *R_b_* is decreased with increasing salt quantity. In any quantity of salt, the density of ions increases; therefore, the *R_b_* decreases considerably. This is the pinpoint of a good agreement with the FTIR results shown in previous section. All the values of DC conductivity were calculated from the bulk resistance and presented in Table 2. It is likely that the incomplete semicircle at a relatively high quantity of salt completely disappeared and the *CPE_2_* component and the *R_b_* are connected in a series, as exhibited in Figure 2b–e. The disappearance of the semicircle at the high frequency is due to the entire movement of ions from the bulk of the electrolyte to the electrode/electrolyte interfaces. This is an indication of the resistive behavior of SPEs. The elements of the equivalent circuit of the blend of electrolytes are listed in Table 1. To interpret this response of the semicircle disappearing In the high-frequency region, the whole spectra reflects the entire conductivity, originating from a huge ion migration [33,39,40,41]. Therefore, the values of *Z_r_* and *Z_i_*, in the EEC design, can be expressed as follows:

(4) $$Z_{r}=\frac{\cos\left(\frac{\pi p}{2}\right)}{Q\omega^{p}}+R_{b}$$

(5) $$Z_{i}=\frac{\sin\left(\frac{\pi p}{2}\right)}{Q\omega^{p}}$$

### 2.3. Dielectric Properties

#### 2.3.1. The Dielectric Constant and Loss Study

**Figure 3:**
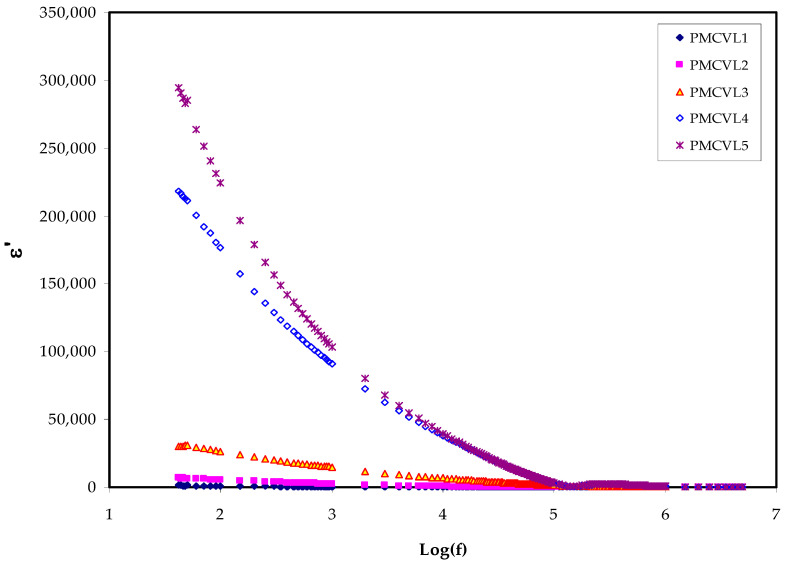
Dielectric constant v Log(f) for all electrolyte films.

**Figure 4:**
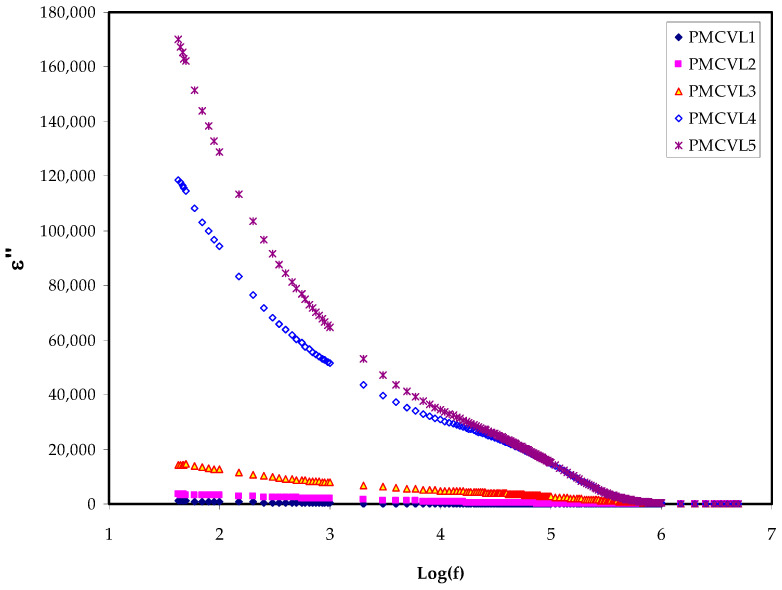
Dielectric loss v Log(f) for all electrolyte films.

To surmount the impact of the space charge polarization, mostly at the electrode/SPE interface, it is necessary to compute conductivity using CI, i.e., by an AC electric field (EF) [42]. The basis of the responses of dielectric spectroscopy involves ion tendencies and dipoles to orient themselves corresponding to an EF [43]. The perturbation of a parallel plate capacitor is separated from polymetric materials by an AC EF, resulting in four categories of polarizations, namely: electronic, atomic, dipolar, and migrating charge polarization, which are in progress [44]. It is often considered that ions act as an impurity in a raw material. In fact, the dipolarity in molecules comes from atoms with largely different electronegativities. This difference in electronegativity exists on the backbone of polymers [43]. The dielectric relaxation mechanisms are normally related to one or more polarization mechanisms of the materials. There are two major contributions in the dielectric response of polymers: namely, the polarization from the dipolarity, and the polarization due to migrating charges. Figure 3 and Figure 4 demonstrate the variation of dielectric constant and dielectric loss as a result of salt concentration changes. Equations (13) and (14) are used to determine the dielectric constant and dielectric loss, respectively. Noticeably, *ε*’ and *ε*″ increase with increasing salt concentrations; in other words, the addition of salt at different concentrations leads to an increase in the dielectric constant and loss at low frequencies. The increase in *ε*′ can be correlated to the ascendance of stored charge ions at the interface of the samples. It can also be said that the increase in the dielectric constant reflects the fractional increase in charges within the electrolyte. It can be observed that the maximum achievable dielectric constant is 50 wt.% NH_4_Cl. This is owing to the existence of a relatively high number of free ions. It was concluded that the dielectric constants remained almost the same at the high-frequency region. This is explained based on the fact that the relatively high polar molecules and ions cannot orient themselves in the SPE under the influence of the high electric field. Therefore, the dielectric constant and its loss keeps its value almost constant with an increasing electric field, as depicted in Figure 3 and Figure 4.

Dielectric measurements (ε*), including the dielectric loss (*ε*″), and the dielectric constant (*ε*′) offer approaches into the chemical and structural behaviors of polymers. These properties are significantly influenced by the addition of another polymer, or a dopant, into the parent polymer [45]. Indeed, in the intensive study of dielectric parameters, the interfacial and electrode polarization of a polymer is of vital importance [46]. The clear observation is that the values of the dielectric parameters gradually increased by increasing the amount of salt, resulting in an increase in the conductivity. This is because of the charge space impact, as well as obeying the non-Debye process in the nature of the conductors [19,47]. It is intuitive that the dielectric constant (*ε*′) and the number density of the charges (*n_i_*) are intimately correlated with each other, as shown in Equation (6) [48]:

*n_i_* = *n_o_* exp (−*U*/*ε*′*KBT*) (6)

where *n_o_*, *U*, *T*, and *K_B_* represent the constants of pre-exponentials, energy dissociation, the absolute temperature, and the Boltzmann constant, respectively. Similarity, DC conductivity indicates the *ε*′ increment. It is well-defined that the mobility (*µ_i_*) and the *n_i_* are two decisive factors (*σ* = *Σqn_i_µ_i_*) that determine the whole conductivity of a given system, where *q* is the charge of the carriers [5,49]. All these confirm that dielectric constant dealing is informative, leading to a comprehensive understanding of the electrical properties of PEs. This shows the extent of the difficulty of ion transport in the PE systems, where there is an interconnection between DC conductivity and ε′ [46,50]. Importantly, establishing an interrelation between DC conductivity and ε′ is obtained via EIS analysis.

#### 2.3.2. Electric Modulus Study

**Figure 5:**
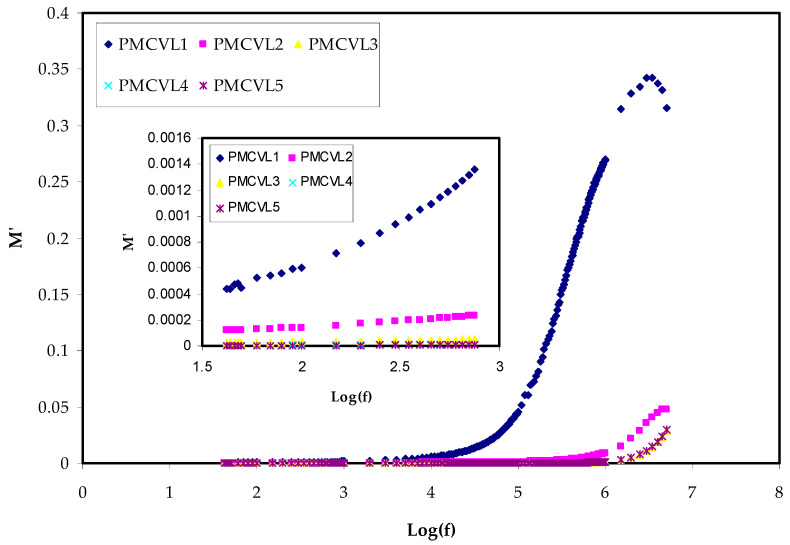
*M*′ v Log(f) for all electrolyte films.

**Figure 6:**
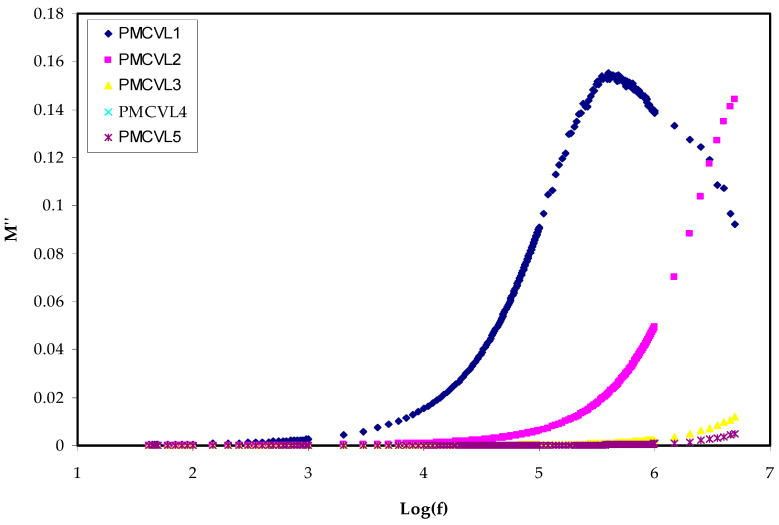
*M*″ v Log(f) for all electrolyte films.

Several other measured or derived quantities can be obtained from impedance spectroscopy (IS). The measurement, analysis, and plotting of some, or all, of the four impedance-related functions (Z*, Y*, M*, and ε*) in the complex plane can be gained from impedance spectroscopy [51]. To have knowledge about the conductive effects and the expression of the complex electric modulus (M* = 1/ε*) is helpful [52]. One of hot topics in the field of condensed matter physics is the study of conductivity relaxation behaviors in conducting polymer materials. This is a result of the many applications in electrochemical devices [53,54]. Examining he relaxation performance of ion-conducting polymers can accurately be performed by means of an electric modulus (M*) formalism. This provides an opportunity to deal with the relaxation and conductivity in a polymer via the electric modulus spectrum [55,56]. The region where the electrical relaxation phenomena occur in PEs is the interface, in addition to the polarization and the phase transition or conductivity mechanism [57]. Equations (16) and (17) are used to determine the *M*′ and *M*″, respectively. The step-like transitions from a low value to a high value in *M*′ spectra are followed (Figure 5) in all the samples. These transitions show the existence of a relaxation mechanism, accompanied by a loss peak in Figure 6 [57]. The peak in *M*″ spectra (Figure 6), which is distinct, can be correlated with conductivity relaxation. Furthermore, an increase in salt concentration results in the shifting of the peaks to the higher-frequency range.

Basically, the appearance of a peak in the imaginary portion *M*″ indicates the region where the charges move at a long displacement (to the left of the peak) or the carriers are confined (to the right of the peak) [58]. As confirmed in earlier studies, the *ɛ*″ parameter is influenced by an ohmic conduction (DC conductivity) [59,60]. Thus, the loss peaks disappear largely in *ɛ*″ spectra, as exhibited in Figure 4.

### 2.4. TNM and LSV Studies

The benign nature of electrolytes is greatly important. The SPEs are essential when used in electrochemical energy devices [61,62]. Despite the familiarization with SPEs since the late 1970s, it is still considered as one of the hot topics of research [21,63].

The electrochemical characterizations of SPEs, via the determination of the TNM and LSV, lead to the decision of whether it is eligible for wide applications or not.

**Figure 7:**
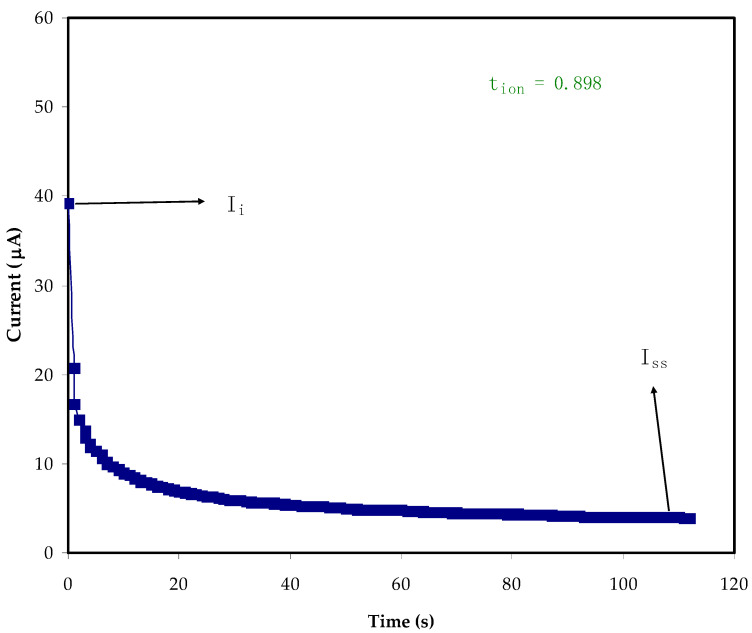
Polarization curve of current for the sample incorporated with 40 wt.% NH_4_Cl.

**Figure 8:**
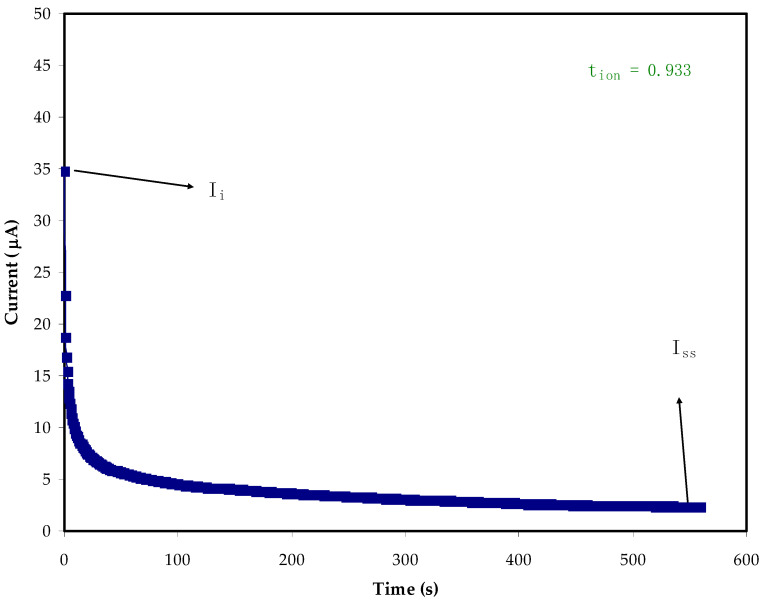
Polarization curve of current for the highest conducting sample (PMCVL5).

To decide on the main charge carrier within the electrolytes, it is essential to find TNM. The DC polarization technique is one of the ways for determining the ion transference number (*t_ion_*) of PE samples. Accordingly, DC voltages are swept, and the currents are recorded correspondingly within an experimental time scale in an electrochemical cell sample at a certain potential window [64]. As stated by Shukur et al. [65], the transference number of the ion (*t_ion_*) is a suitable indicator of when it is in unity, as ions are regarded as the dominant charge carrier within the PE systems. Figure 7 and Figure 8 reveal the steady state, during polarization, for the PVA:MC:NH_4_Cl. The current decreases at the beginning and as the time proceeds, there is a current decay where the ionic species depletion within the electrolytes, and the diffusion process are responsible for mobile ion movement [66,67]. The initial high-value current is because the ions and electrons are participating in the beginning. The polarization of the cell occurs when it reaches the steady state, while the moving of the rest of the current is only due to electrons. This is due to the fact that the stainless-steel electrodes make a barrier for the ions, while only allowing the electrons to move through [41]. This is why the electron transference number (*t_ele_*) is measured by the stainless-steel electrodes [68]. Equations (7) and (8) are used to determine *t_ion_* and *t_ele_*, respectively [69,70]. In this work, the average value of *t_ion_* for the electrolyte (PMCVL5) is 0.933, while the *t_elec_* for (PMCVL4) is 0.088, supporting the idea that ions are the dominant charge carriers in the PVA:MC:NH_4_Cl electrolyte. This behavior can be exploited for utilization in electrochemical energy devices. The glycerolized chitosan–NH_4_Br system has also previously been reported to have comparable results [71].

(7) $$t_{ion}=\frac{I_{i}-I_{ss}}{I_{i}}$$

(8) $$t_{ele}=1-t_{ion}$$

In these equations, the initial and the steady state current are denoted as *I_i_* and *I_ss_*, respectively.

**Figure 9:**
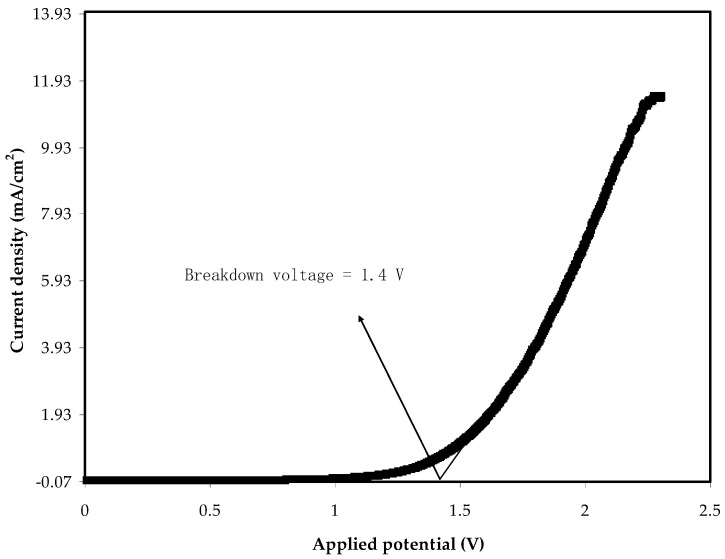
LSV plot for the PMCVL5 system.

The linear sweep voltammetry (LSV) tells us the maximum operating voltage of an electrolyte, where there is a range that allows for the implemented voltages to work [72]. Figure 9 shows the LSV response of the electrolyte at 10 mV/s at room temperature. In the LSV, the applied voltage varied from 0 to 2.5 V. From 0 to below 1.4 V, the current did not pass through the electrolyte, meaning that the sample can withstand below 1.4 V. While electrolysis occurs beside the 1.4 V and the current passes through the PE due to the occurrence of oxidation. As mentioned above, below 1.4 V, no current flows within the electrolyte, indicating the absence of any electrochemical reaction [73]. The decomposition of the PVA:MC:NH_4_Cl, as the electrolyte of use, at the voltage of 1.4 V, occurs. Consequently, this voltage range makes this electrolyte eligible for its utilization in EDLC, which exceeds the normally operating voltage of 1.0 V [74]. Here, the voltage window range of the PE is 1.4 V, which is reasonable.

### 2.5. Cyclic Voltammetry Study

**Figure 10:**
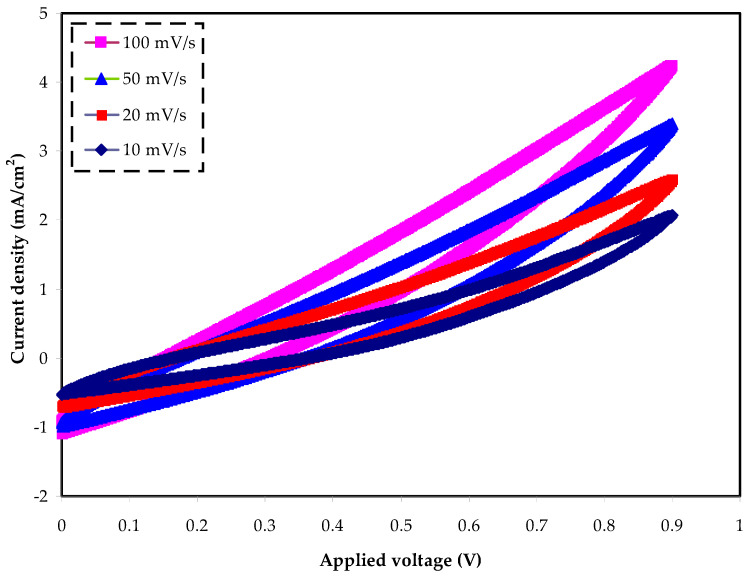
Cyclic voltammetry (CV) at various scan rates of 10, 20, 50, and 100 mV/s.

From the cyclic voltammogram, one can recognize the charge storage behavior at the electrodes–electrolyte interface of an EDLC assembly. The CV was performed for the electrolyte at different scan rates, as revealed in Figure 10. It is seen that the CV response distorts clearly from th rectangular shape to the sharp leaf shape. This phenomenon originated from two parameters: namely, the carbon electrode’s porosity, and the internal resistance [75]. Especially at higher scan rates, the CV curves are very close to a leaf shape, as at higher scan rates the ions move very fast, and no proper double-layers are formed at the electrode–electrolyte interfaces. From Figure 10, it is obvious that there are no significant differences in the double-layer charging current with varying scan rates. To our knowledge, the lattice energy (LE) of salts is affect greatly by the electrochemical behavior of the devices. Salts with a high LE allow little ions to dissociate and contribute to the formation of a double layer of charges. The LE of NH_4_Cl, at 708 KJ/mol, is higher than that of 682 KJ/mol for NH_4_Br, or 637 KJ/mol for NH_4_I salt [76]. Moreover, there are no peaks in the allowed voltage range of the CV, indicating that neither oxidation nor reduction in the EDLC occurs. Principally, the charging mechanism causes anions and cations to move oppositely toward the anode and cathode electrodes, respectively, in the EDLC assembly. The negatively charged electrode (cathode) will attract positive ions (cations) from the PE solution, while the positively charged electrode (anode) will attract negative ions (anions) from the electrolyte solution. The stainless-steel electrodes block ions at the surface of the electrodes to form a double-layer capacitor at the surface of the electrodes. The induced electrical field leads to an interaction between the anode (positive) electrode and the anions; in contrast, the opposite situation takes place at the negative (cathode) electrode. The relatively high electric field results in the electrons and ions to be held by electrode and electrolyte, respectively [77]. This is the double-layer charge formation, which is developed on the surface of carbon electrodes where the stored energy is in the form of potential energy [78]. The values of *C_s_* are calculated by using Equation (9) [79] at various scan rates, as tabulated in Table 3. However, by adding a plasticizer, for example, glycerol, to the PE system, the values of *C_s_* can be improved, as glycerol can dissociate more salts into free ions and, thus, more ions are adsorbed at the surface of the electrodes [41]:

(9) $$C_{spe}=\int_{V_{i}}^{V_{f}}\frac{I\left(V\right)dV}{2mv\left(V_{f}-V_{i}\right)}$$

where *I(V)dV* represents the *CV* area, which can be gained from Origin 9.0 software via the integration function, and the working voltage, *V_i_* and *V_f_*, are 0 and 0.9 V, respectively. The *m* and *v* stand for the mass of activated carbon electrode materials and the scan rate, respectively. Generally, the *C_s_* values decrease significantly as the scan rate increases. This is due to decreasing the number of stored charges on the electrode surface at the high scan rate which, in turn, led to the energy loss increase [80].

## 3. Materials and Methods

### 3.1. Materials and Electrolyte Preparation

All raw materials in this research were received from Sigma-Aldrich and were used as obtained in preparation with no purification. Zero point four grams of PVA was dissolved in distilled water at 80 °C. At the same time, 0.6 g of MC was dissolved in 50 mL of 1 wt.% acetic acid for 1.5 h at room temperature. Subsequently, the solutions of PVA and MC were consistently mixed for 3 h. Then, various quantities of NH_4_Cl from 10 wt.% to 50 wt.% were loaded into the solution of the PVA–MC that agitated constantly until the salt dissolved entirely, as presented in Table 4.

In the preparation of the PE film, the solution casting process was implemented. Accordingly, the prepared PE samples were cast in Petri dishes and were left to dry. To ensure dryness, the films were left in a desiccator using blue silica gel at room temperature. All preparation processes occurred at room temperature with 50% relative humidity. The PE samples possessed a thickness within a range of 0.028 to 0.031 cm and were then evaluated via a high-accuracy micrometer (Mitutoyo, Coventry, UK).

### 3.2. Electrical Impedance Spectroscopy (EIS)

Complex impedance spectroscopy is one of reliable techniques that can be used in dealing with electrical properties of materials, especially at the interfaces. From the data point analysis obtained, one can obtain insight into the electrical properties of the materials and the interfaces. The a.AC conductivity is principally gained from the cell impedance/admittance measurements over a range of temperatures and frequencies. The concept of impedance is more accurate than resistance, because it takes into account the phase differences. Based on the AC, the resistance term, *R*, is replaced by the impedance, *Z* (where *Z* is the sum of the resistance and reactance) [81].

Prior to the measurements, the SPE films were cut into small discs, which are 2 cm in diameter and were then fixed between two stainless-steel electrodes, which were pressed by a spring. The stainless-steel electrodes were used to block ions at the surface of the electrodes to create double layer capacitor at the surface of the electrodes. The HIOKI 3531 Z Hi-tester was hyphenated to a computer that was used in the measuring of the conductance of the films in the frequency range of 50 Hz to 5000 kHz at room temperature. The software controlled the measurements and calculated the real and imaginary parts of the impedance. It is well-known that from the real (*Z*′) and imaginary (*Z*″) parts of complex impedance (Z*), the real and imaginary parts of permittivity (ε*) and the modulus (M*) can be obtained using the following mathematical relationships shown below [21,82]:

(10) $$Z^{*}=Z^{\prime }-jZ^{\prime \prime }$$

(11) $$\varepsilon^{*}=\varepsilon^{\prime }-j\varepsilon^{\prime \prime }=\frac{1}{j\omega \varepsilon_{o}Z^{*}}$$

(12) $$M^{*}=1/\varepsilon^{*}=j\omega C_{o}Z^{*}=M^{\prime }+jM^{\prime \prime }$$

From Equations (10)–(12), one can obtain the following relationships:

(13) $$\varepsilon^{\prime }=\frac{Z^{\prime \prime }}{\omega C_{o}({Z^{\prime }}^{2}+{Z^{\prime \prime }}^{2})}$$

(14) $$\varepsilon^{\prime \prime }=\frac{Z^{\prime }}{\omega C_{o}({Z^{\prime }}^{2}+{Z^{\prime \prime }}^{2})}$$

(15) $$Tan\delta =\frac{\varepsilon^{\prime \prime }}{\varepsilon^{\prime }}$$

(16) $$M^{\prime }=\frac{\varepsilon^{\prime }}{\left({\varepsilon^{\prime }}^{2}+{\varepsilon^{\prime \prime }}^{2}\right)}=\omega C_{o}Z^{\prime \prime }$$

(17) $$M^{\prime \prime }=\frac{\varepsilon^{\prime \prime }}{\left({\varepsilon^{\prime }}^{2}+{\varepsilon^{\prime \prime }}^{2}\right)}=\omega C_{o}Z^{\prime }$$

where, *C_o_* reflects the vacuum capacitance (*ε_o_A*/*t*), and *t* and *A* represent the thickness and the area of each sample, respectively. The angular frequency *ω* equals to *ω = 2πf,* where *f* is the applied frequency.

Fourier transform infrared (FTIR) spectroscopy can provide evidence between interacting elements of the electrolytes, for example, salts and polymers. For this purpose, a Perkin–Elmer Spotlight 400 spectrometer was employed, possessing a 1 cm^−1^ resolution (450 cm^−1^–4000 cm^−1^). To have distinct peaks, the deconvolution method was implemented.

### 3.3. Transference Number Measurement (TNM) and Linear Sweep Voltammetry (LSV) Measurement

Transference number measurement (TNM) was used to tackle the electrochemical properties of the SPEs by a V&A Instrument (DP3003) (Shanghai, China) coupled to a digital DC power source. Previously, [46], the circuit for the TNM study was designed. From the TNM, both the ion transference (*t_ion_*) and the electron transference numbers (*t_e_*) were measured. At room temperature, the working voltage was held constant at 0.20 V. Moreover, the electrochemical stability of the PE, showing the breakdown voltage, is of great importance. Therefore, the LSV was obtained using a potentiostat (DY2300) (Neware, Shenzhen, China) at various sweep rates of 10, 20, 50, and 100 mV/s. For this purpose, the three electrodes system which are working electrode, counter electrode, and reference electrode were used.

## 4. Conclusions

In summary, the implementation of the solution casting method was performed in fabricating SPEs containing NH_4_^+^ as the conducting species. The molecular interaction between the components of the electrolyte was confirmed via the frequency of the bonds from the FTIR spectra. The circuit designs for each system were obtained from the simulation of EIS data. The dielectric response technique was applied to determine the extent of ion dissociation of the NH_4_Cl in the PVA–MC–NH_4_Cl systems. The trend of *ɛ*′ was found to be similar to the trend of σ_dc_, with a salt concentration. The appearance of an asymmetric peak in the imaginary part of modulus study recognizes the occurrence of coupling between chain dynamics and ion mobility. The electrolyte possesses satisfied conductance via the addition of proper salt, and the double layer formation, as a way of charging the storing mechanism, was verified from the CV shape. Transference number measurement (TNM) was found to be (*t_ion_*) = 0.933 for the highest conducting film. This proves that the ions are the most dominant charge carriers. From the linear sweep voltammetry (LSV) study, 1.4 V was measured for the maximum conducting sample. The cyclic voltammetry response appeared in the form of a sharp leaf shape, rather than a redox peak with a specific capacitance of 20.6 F/g at 10 mV/s. The electrochemical voltage window of the prepared polymer electrolyte indicates the eligibility of the electrolyte for its utilization in the EDLC device.

## Figures and Tables

**Table 1:** EEC fitting parameters for the electrolyte systems.

Designation	K_1_ (F^−1^)	K_2_ (F^−1^)	C_1_ (F)	C_2_ (F)
PMCVL1	1.60 × 10^8^	1.70 × 10^6^	6.25 × 10^−9^	5.88 × 10^−7^
PMCVL2		2.15 × 10^6^		4.65 × 10^−7^
PMCVL3		5.90 × 10^5^		1.69 × 10^−6^
PMCVL4		6.90 × 10^4^		1.45 × 10^−5^
PMCVL5		4.20 × 10^4^		2.38 × 10^−5^

**Table 2:** DC conductivity and *R_b_* values for the electrolyte samples.

Designation	DC Conductivity (S/cm)
PMCVL1	1.02 × 10^−6^
PMCVL2	9.62 × 10^−5^
PMCVL3	1.49 × 10^−4^
PMCVL4	1.03 × 10^−3^
PMCVL5	1.99 × 10^−3^

**Table 3:** Specific capacitance value against scan rates.

Scan Rate	V2-V1	Capacitance (F/g)
0.1	0.9	4.161
0.05	0.9	7.773
0.02	0.9	15.775
0.01	0.9	20.645

**Table 4:** Designation of the PEs with different amounts of salt.

PVA–MC–NH_4_Cl (wt.%)	Designation
40-60-10	PMCVL1
40-60-20	PMCVL2
40-60-30	PMCVL3
40-60-40	PMCVL4
40-60-50	PMCVL5

## Data Availability

Not applicable.
